# Current Biomarkers for Carotid Artery Stenosis: A Comprehensive Review of the Literature

**DOI:** 10.3390/metabo13080919

**Published:** 2023-08-05

**Authors:** Hamzah Khan, Farah Shaikh, Muzammil H. Syed, Muhammad Mamdani, Gustavo Saposnik, Mohammad Qadura

**Affiliations:** 1 Division of Vascular Surgery, St. Michael’s Hospital, Toronto, ON M5B 1W8, Canada; hamzah.khan@mail.utoronto.ca (H.K.); farah.shaikh@unityhealth.to (F.S.); muzammil.syed@mail.utoronto.ca (M.H.S.); 2 Li Ka Shing Knowledge Institute, St. Michael’s Hospital, Unity Health Toronto, Toronto, ON M5B 1W8, Canada; muhammad.mamdani@unityhealth.to (M.M.); gustavo.saposnik@unityhealth.to (G.S.); 3 Temerty Centre for Artificial Intelligence Research and Education in Medicine (T-CAIREM), University of Toronto, Toronto, ON M5S 1A1, Canada; 4 Division of Neurology, Department of Medicine, St. Michael’s Hospital, University of Toronto, 55 Queen St E, Toronto, ON M5C 1R6, Canada; 5 Department of Surgery, University of Toronto, Toronto, ON M5T 1P5, Canada

**Keywords:** carotid, stenosis, carotid artery stenosis, atherosclerosis, biomarkers, proteins, cardiovascular

## Abstract

Carotid artery stenosis (CAS), an atherosclerotic disease of the carotid artery, is one of the leading causes of transient ischemic attacks (TIA) and cerebrovascular attacks (CVA). The atherogenic process of CAS affects a wide range of physiological processes, such as inflammation, endothelial cell function, smooth muscle cell migration and many more. The current gold-standard test for CAS is Doppler ultrasound; however, there is yet to be determined a strong, clinically validated biomarker in the blood that can diagnose patients with CAS and/or predict adverse outcomes in such patients. In this comprehensive literature review, we evaluated all of the current research on plasma and serum proteins that are current contenders for biomarkers for CAS. In this literature review, 36 proteins found as potential biomarkers for CAS were categorized in to the following nine categories based on protein function: (1) Inflammation and Immunity, (2) Lipid Metabolism, (3) Haemostasis, (4) Cardiovascular Markers, (5) Markers of Kidney Function, (6) Bone Health, (7) Cellular Structure, (8) Growth Factors, and (9) Hormones. This literature review is the most up-to-date and current comprehensive review of research on biomarkers of CAS, and the only review that demonstrated the several pathways that contribute to the initiation and progression of the disease. With this review, future studies can determine if any new markers, or a panel of the proteins explored in this study, may be contenders as diagnostic or prognostic markers for CAS.

## 1. Introduction

Atherosclerotic disease affects hundreds of millions of people worldwide, and remains to be the leading cause of death globally, contributing to 31% of all deaths in 2015 [1,2,3]. Atherosclerosis occurs when there are increased levels of cholesterol, such as low density lipoprotein (LDL), within circulating blood, which eventually builds up within the subendothelial space of the vasculature [4,5,6]. This leads to endothelial damage and the migration of inflammatory cells, such as T-cells and monocytes into the intimal layer of the endothelium [7]. The infiltrating monocytes differentiate into macrophages and take up excess lipids, converting into foam cells. These foam cells eventually undergo apoptosis and necrosis, leading to further enhancement of the immune response [8]. The immune response triggers the release of pro-inflammatory mediators that recruit further inflammatory cells to the area. These mediators also lead to the proliferation and migration of vascular smooth muscle cells (VSMC) and the deposition of extracellular matrix proteins within the area [9]. This vicious cycle continues until there is large atherosclerotic plaque that has become calcified with a fibrous cap. This plaque can lead to significant stenosis of the artery and poses the risk of rupture and thrombus formation, which, in some cases, may dislodge and lead to downstream arterial stenosis.

A common location of atherosclerotic plaque formation is within the bifurcation of the common carotid artery, where the artery first branches into the internal and external carotid, and is known as carotid artery stenosis (CAS). CAS can occur up or downstream in either artery [10,11] and is known to cause approximately 15–20% of cerebrovascular attacks (CVA)—a major contributor of patient disability and death [12,13]. Guidelines recommend medical management with antiplatelets, smoking cessation, and treatment of hypertension and hypercholesterolemia for CAS patients. With regard to surgical intervention, carotid endarterectomy (CEA) is a recommended first-line treatment for the primary and secondary prevention of CVA for most symptomatic and asymptomatic patients [14], whereas carotid artery stenting is only recommended for patients with symptomatic CAS with stenosis > 50%, provided CEA is not a feasible option.

Recent literature, however, suggests that early medical management may be as effective, if not more, at preventing CVAs than surgical intervention in a some CAS patients [15]. In this regard, biomarkers may play a crucial role in assisting with both early diagnosis and treatment initiation, as well as risk stratification for determining which patients are at increased risk of adverse events, and who may benefit from surgical interventions. Currently, no such biomarkers have been discovered that have strong diagnostic or prognostic capability and have been proven to be clinically relevant. Hence, the discovery of a clinically relevant biomarker for the purpose of diagnosing patients with CAS and predicting adverse outcomes in this patient population is required.

Atherosclerotic lesions contain a plethora of cells, proteins and metabolites within plaque, including immune cells, cholesterol, fatty acid transport proteins among many others [16]. These molecules and cells often diffuse into the blood, and levels can be detected in plasma. In this literature review, we will focus on the current research on diagnostic and prognostic blood protein biomarkers for CAS and categorize them based on their primary physiological function in the body. By identifying current markers for CAS and exploring physiological contributions of the proteins, this allows us to obtain a better understanding of overall disease initiation and progression, and may inform which proteins are best for diagnosis/prognosis of CAS. Given the surge of interest surrounding the integration of machine learning models for diagnostics and risk stratification in medical research, this review may also assist in determining which proteins would be best used as parameters in such models of CAS.

## 2. Methods and Literature Review

The MEDLINE database was searched for current research (January 2010–August 2022) evaluating the plasma/serum protein biomarkers, carotid plaque and adverse outcomes in patients with carotid artery stenosis in humans. The terms carotid artery stenosis and biomarkers were cross searched in the database with filters set to research past January 2010. Two researchers conducted the literature review independently in order to prevent missing articles.

**Figure 1:**
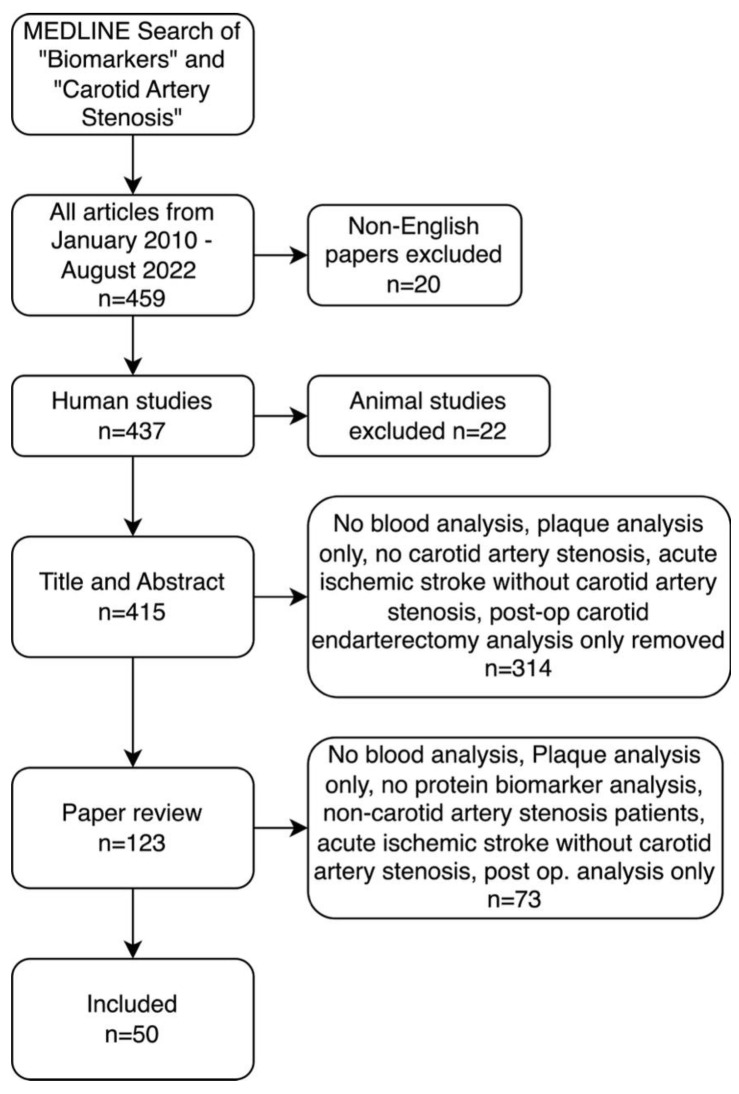
Preferred Reporting Items for Systematic Reviews and Meta-Analyses (PRISMA) diagram for MEDLINE search methodology for the inclusion and exclusion criteria of articles included in literature review.

Of the 479 articles yielded by the search, 20 non-English articles and 22 non-human studies were excluded. Titles and abstracts, as well as whole texts, were evaluated. Research studies that focused solely on the following were excluded: non-blood biomarkers, biomarkers other than proteins, plaque immunohistochemistry only, non-ischemic stroke, ischemic stroke without carotid artery stenosis and post CEA assessment only. These were excluded to ensure only studies focusing on blood biomarkers in patients with atherosclerotic carotid artery disease (Figure 1).

From the final 50 studies of the review (Table 1), a total of 36 proteins were found as potential biomarkers for CAS and were categorized into the following nine categories based on primary protein function: (1) Inflammation and Immunity, (2) Lipid Metabolism, (3) Haemostasis, (4) Cardiovascular Markers, (5) Markers of Kidney Function, (6) Bone Health, (7) Intracellular Structure, (8) Growth Factors and (9) Hormones (Figure 1).

## 3. Protein Categorization

**Figure 2:**
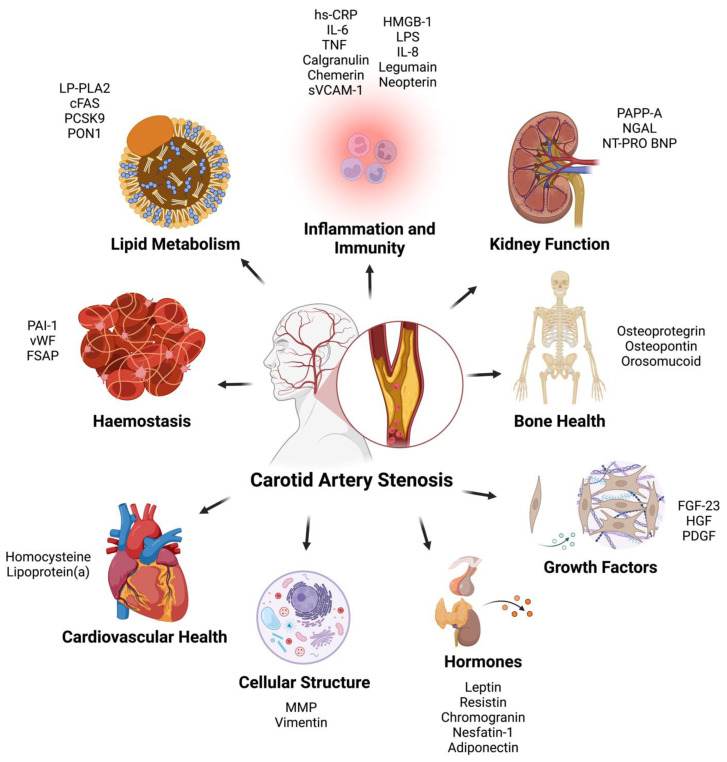
Carotid artery stenosis biomarkers categorized based on primary function. hs-CRP = High Sensitivity—C Reactive Protein; IL-6 = Interleukin-6; TNF = Tissue Necrosis Factor; sVCAM-1 = Soluble Vascular Cell Adhesion Molecule-1; HMGB-1 = High Mobility Group Box-1; LPS = Serum Lipopolysaccharide-Binding Protein; IL-8 = Interleukin-8; PAPP-A = Pregnancy-Associated Plasma Protein A; NGAL = Neutrophil Gelatinase Associated Lipocalin; NT-PRO BNP = N-Terminal Pro B-Type Natriuretic Peptide; FGF-23 = Fibroblast Growth Factor—23; HGF = Hepatocyte Growth Factor; PDGF; Platelet Derived Growth Factor; MMP = Matrix Metalloproteinases; PAI-1 = Plasminogen Activator Inhibitor-1; vWF = Von Willebrand Factor; FSAP = Factor VII Activating Protease; LP-PLA2 = Lipoprotein-Associated Phospholipase A2; cFAS = Circulating Fatty Acid Synthase; PCSK9 = Proprotein Convertase Subtilisin/Kexin Type 9; PON1 = Paraoxonase-1.

All biomarkers yielded from the literature review were grouped into one of nine categories: (1) Inflammation and Immunity, (2) Lipid Metabolism, (3) Haemostasis, (4) Cardiovascular Markers, (5) Markers of Kidney Function, (6) Bone Health, (7) Cellular Structure, (8) Growth Factors and (9) Hormones. Categorizations were based on the primary function of each protein (Figure 2).

### 3.1. Inflammation and Immunity

The immune response, and its associated inflammatory response, is a well-known indicator of CAS and serves as the initiating factor in the development and progression of atherosclerotic disease [67]. Inflammation is often triggered by the accumulation of oxidative low-density lipoprotein in the arterial wall, which leads to injury of the endothelial lining of the vasculature and activation of immune cells [5]. Reducing inflammation is a key aspect of the management of CAS in order to prevent the progression of the disease and its associated adverse outcomes. The following eleven inflammation and immunity-related proteins demonstrated strong suitability as potential inflammatory biomarkers for CAS.

#### 3.1.1. High Sensitivity—C Reactive Protein

High Sensitive C-Reactive protein (hs-CRP) is a commonly cited potential biomarker for CAS within the literature. CRP is an acute phase protein that is released from the liver in response to several different inflammatory cytokines [68]. Since inflammation increases before the onset of stroke, CRP has been thoroughly investigated in its use as a biomarker for CAS [69]. Perl et al., in 2016, demonstrated in 522 patients that increasing severity of CAS was associated with increased hs-CRP levels [39]. Similarly, other studies also reported increased levels of CRP in CAS patients when compared to controls [19,33,40,58,66]. A few studies also demonstrated that CRP was higher in patients with unstable plaque and in patients with neurological symptoms when compared to stable plaques and asymptomatic patients, respectively [38,47,48,66]. Some researchers did not observe a significant difference between symptomatic and asymptomatic patients, though small sample sizes were utilized in these investigations [40,43]. In terms of prognostication, higher CRP levels have been associated with higher rates of neurological events [49,57]. Currently, the American Heart Association and the American College of Cardiology recommends routine CRP testing only when a treatment decision after quantitative risk assessment remains unclear.

#### 3.1.2. Interleukin-6

Interleukin-6 (IL-6) is a soluble plasma glycoprotein and an inflammatory mediator primarily produced by monocytes [70]. It is released in response to cellular injury and infections [71]. IL-6 was demonstrated to be predictive of CAS [66,72], with IL-6 being significantly higher in patients with symptomatic CAS compared to asymptomatic patients (4.3 ± 1.7 vs. 3.3 ± 1.1 pg/dL, respectively, *p* = 0.017) [43]. A few studies also noted it to be higher in patients with plaque instability, potentially demonstrating higher levels of local inflammation [47,66]. One study, however, noted no association between IL-6 and the degree of stenosis [66].

#### 3.1.3. Tissue Necrosis Factor

Tissue Necrosis Factor (TNF) is an inflammatory cytokine produced by immune cells, and plays a variety of important roles in many cellular functions, including cell differentiation, proliferation and apoptosis [73]. A previous study demonstrated upregulated levels of TNF in vulnerable plaques; however, this was not correlated with increased levels within plasma [47].

#### 3.1.4. Calgranulins

The calgranulin family compromises of three proteins that have important roles in the regulation of inflammation: calgranulin A (S100A8), calgranulin B (S100A9) and calgranulin C (S100A12). However, they also have anti-oxidant and infection protective properties [74]. They can be found within squamous mucosal keratinocytes and immune cells located on mucosal surfaces, and function through the chelation of manganese and zinc. The reduction in these cation levels reduces the reproducibility of some bacteria and fungi [75]. A recent study noted that levels of all three aforementioned calgranulins were three-fold higher (*p* < 0.001) in patients with CAS when compared to controls. The authors also noted that calgranulins A and B were highest in the group with the most recent symptom onset [37].

#### 3.1.5. Chemerin

Chemerin is a chemoattractant, inflammatory cytokine, also known as retinoic acid receptor responder 2 (RARRES2) [76]. It is primarily produced by the liver and white adipose tissues, and secreted into plasma as pro-chemerin [77]. It can be cleaved by several tissues into its chemoattractant form (chemerin), and can interact with several different proteins with a wide variety of functions from skin turnover, metabolism, angiogenesis, to adipogenesis [78]. A study conducted by Kammerer et al. in 2018 demonstrated that chemerin was significantly increased in patients with CAS by 1.3 fold (*p* < 0.001), and was correlated with other inflammatory markers such as CRP (*p* < 0.001). In patients with CAS greater than 90%, chemerin was increased in symptomatic patients compared to asymptomatic patients (*p* = 0.014). The researchers noted that increasing levels of chemerin significantly correlated with an increase in the risk of coronary artery disease (odds ratio = 1.002 [1.001–1.003], *p* = 0.001). [33]. Interestingly, a different study conducted by Gasbarrino et al. demonstrated that chemerin was negatively correlated with plaque instability, with increasing chemerin in plasma reducing the odds of unstable plaque [51]. They also noted a three-fold reduction in the odds of having unstable plaque when comparing the first and third tertiles of plasma chemerin concentration [51]. The authors suggested that chemerin has both inflammatory and anti-inflammatory properties, which may explain the contrasting results.

#### 3.1.6. Soluble Vascular Cell Adhesion Molecule-1 

Vascular Cell Adhesion Molecule-1 (VCAM-1) is a protein that is expressed on vascular endothelial cells in response to the release of tumor necrosis factor alpha (TNFα) by macrophages/monocytes, and functions by facilitating the adhesion and transendothelial migration of leukocytes to local areas of inflammation [79]. Once cleaved and released into the blood stream, it is known as soluble VCAM-1 (sVCAM-2). sVCAM-2 has been well studied for its potential as a biomarker several diseases, such as cardiovascular disease, immunological disease, autoimmune disease, cancer and many more [80]. Two recent studies investigated its potential as a marker for CAS specifically. The first study was conducted in 2014 by Shindo et al. who investigated if there was an increase in the local release of sVCAM-1 near carotid plaque formation when compared to systemic samples located more peripherally from the plaque [47]. They noted that sVCAM-1 levels were significantly increased in local blood samples collected from the carotid artery when compared to blood collected from the femoral artery (*p* = 007). However, no significant differences were noted in serum levels of sVCAM-1 when comparing CAS patients to controls, and CAS patients with stable versus vulnerable plaque [47]. A second study conducted by Puig et al. in 2021 suggested a significant increase in sVCAM-1 in symptomatic patients with CAS when compared with controls, and that increased plasma sVCAM-1 was independently associated with carotid plaque inflammation (β  =  0.144, 95% CI 0.012–0.276, *p*  =  0.033) [52].

#### 3.1.7. High Mobility Group Box-1

High mobility group box-1 (HMGB1) is a non-histone chromosome-binding protein that has multiple functions based on the location and state of the cell [81]. It can function as both a transcription regulator and as a damage-associated molecular pattern molecule [82]. When HMGB1 is found within the nucleus, it functions by regulating gene transcription and DNA organization [83]. When a cell is injured, HMGB1 is translocated to the cytoplasm, or released from the cell, where it stimulates inflammation and cellular repair mechanisms [30,82]. It can stimulate macrophages to release Tissue Necrosis Factor (TNF) and Interleukin-6 to drive inflammation [82]. A study conducted Biscetti et al. investigated the possibility of using HMGB1 as a marker for CAS in diabetic patients. The researchers demonstrated that HMGB1 was significantly elevated in diabetic patients with CAS compared to diabetic controls (7.65 ± 13.32 versus 2.96 ± 7.45 ng/mL, respectively, *p* < 0.001), and logistic regression determined that HMGB1 was independently associated with unstable plaque [84].

#### 3.1.8. Serum Lipopolysaccharide-Binding Protein 

Lipopolysaccharide (LPS) is a major cell surface glycolipid commonly found in many Gram-negative bacteria [85]. Lipopolysaccharide-Binding Protein (LBP) is a human glycoprotein produced by hepatocytes and endothelial cells of the intestine that initiates an acute-phase reaction and innate immune response when bound to LPS [86]. When LPS binds to LBP, an series of signal transduction pathways are activated, leading to the release of cytokines and pro-inflammatory mediators [87]. Patients that consume high levels of fat and sugar-rich diets were shown to have higher levels of LBP in serum, perhaps indicating why diabetes and obesity may be attributed to having chronic low level inflammation [88,89]. Previous studies demonstrated that elevated levels of LBP were independently associated with coronary artery disease, that LBP was positively correlated with carotid intimal medial thickness and that LBP was significantly higher in patients with CAS compared to healthy controls (32.7 ± 12.5 versus 28.7 ± 10.7, *p* = 0.021) [36].

#### 3.1.9. Interleukin-8

Interleukin-8 (IL-8) is a member of the Interleukin family that includes other small chemotactic peptides with structural homology. It also shares DNA sequence features with other cytokines, indicating common regulatory pathways [90,91]. IL-8 is a chemoattractant cytokine produced by a variety of tissue and blood cells and it has a distinct target specificity for the neutrophil, with only weak effects on other blood cells. Interleukin-8 attracts and activates neutrophils in inflammatory regions through interactions with the endothelial mesothelial interactions. Neutrophils respond to IL-8 by undergoing a series of changes including cellular migration, the release of granule enzymes, as well as other intra- and extracellular changes [90]. The only study so far investigating the IL-8 in CAS was the Sharma G. et al., demonstrating no significant differences in IL-8 levels between symptomatic and asymptomatic CAS patients [21].

#### 3.1.10. Legumain

Legumain is a cysteine protease that can break down certain types of proteins through the hydrolysis of asparagine bonds [92]. It is usually found in lysosomes, where it helps the body recognize and respond to foreign substances through antigen processing and presentation for immune cells [93]. Recent studies suggested that legumain may also play important roles in other processes, such as immunity and cancer [94]. Several studies posited legumain as a marker for unstable atherosclerotic plaque [95,96]. In a study of 254 patients with CAS and 91 controls, researchers determined that legumain levels were higher in patients with CAS when compared to controls (2.0 versus 1.5 ng/mL, respectively; *p* = 0.003). Levels of legumain were also highest in the CAS group of patients with the most recent symptoms [23].

#### 3.1.11. Neopterin 

Neopterin belongs to the class of pteridines and has been identified as an early biomarker of the cellular immune response. Neopterin is produced by activated macrophages and dendritic cells [97]. Increased levels of neopterin in bodily fluids have been associated with different diseases that involve the activation of the cellular immune mechanism; such diseases include certain malignancies, autoimmune diseases, and viral infections [97]. Neopterin has been studied as a potential marker of cardiovascular risk and clinical studies demonstrated a correlation between neopterin levels and peripheral artery disease and CAS [98,99]. Only one recent study concluded that circulating neopterin levels are significantly higher in patients with severe CAS than in those with acute ischemic stroke (*p* < 0.001) [24].

### 3.2. Lipid Metabolism

Lipid metabolism refers to the process by which the body produces, transports, stores and breaks down lipids and fatty acids for metabolism [100]. Dysregulation of this process can have significant effects on the development and progression of CAS. High levels of low-density lipoprotein (LDL) cholesterol can lead to the accumulation of lipids in the arterial walls leading to damage and inflammation, and is a known driving force for atherosclerotic disease [6]. Maintaining a healthy lipid profile through a balanced diet and regular exercise can play a crucial role in preventing carotid artery disease and other cardiovascular conditions, and many markers of lipid metabolism can help diagnose and predict lipid metabolism and lipid profile disorders. Some of these lipid metabolism markers may play key roles in understanding the diagnosis and progression of CAS.

#### 3.2.1. Lipoprotein-Associated Phospholipase A2

Lipoprotein-Associated Phospholipase A2 (Lp-PLA_2_) is a member of the Phospholipase A2 family and is calcium-independent. Its function is the oxidation of low-density lipoprotein (LDL) within the vascular wall. This protein releases oxidized phospholipids such as lysophosphatidylcholine and oxidized non-esterified fatty acids, which are associated with inflammation and the progression of atherosclerosis [101]. Previous studies demonstrated Lp-PLA_2′_s association with coronary artery disease [102]; however, three studies investigated its association with patients with CAS.

A study conducted by Sarlon-Bartoli et al. in 2012 demonstrated that patients with unstable and/or ulcerated plaque had increased Lp-PLA_2_ levels when compared with those with stable plaque morphology (222.4 (174.9–437.5) versus 211.1 (174.9–270.6) ng/mL, respectively, *p* = 0.02) [38]. Stefanic et al. demonstrated significant differences in Lp-PLA_2_ in symptomatic versus asymptomatic patients (*p* < 0.001) [48]. In 2020, researchers noted that in a group of 823 patients with CAS, those in the fourth quartile for Lp-PLA_2_ levels were at a 10.17-fold increased risk of a CVE than those in the first quartile, demonstrating the potential of Lp-PLA_2_ being a strong prognostic marker for CVAs in patients with CAS.

#### 3.2.2. Circulating Fatty Acid Synthase

Circulating fatty acid synthase (cFAS) is an enzyme found within lipogenic tissue that plays a vital role in fatty acid synthesis by producing saturated fatty acids using molecules such as acyl-CoA and malonyl-CoA [103]. Its primary role is the storage of energy for future use in the form of fatty acids when energy supplies are high [104]. High activity of cFAS has been implicated in several cancers, such as colon cancer, ovarian cancer, breast cancer and more [105]. It was also demonstrated to have increased in expression in obesity and diabetes [106]. One study explored the levels of cFAS in patients with CAS patients undergoing CEA, and they found ~5-fold higher levels of cFAS compared to patients without CAS. No differences were noted in symptomatic versus asymptomatic patients. CAS patients with diabetes mellitus are reported to have higher levels of cFAS than non-diabetic CAS patients [31].

#### 3.2.3. Proprotein Convertase Subtilisin/Kexin Type 9

Proprotein Convertase Subtilisin/Kexin Type 9 (PCSK9) is a protease that has an important role in cholesterol regulation via binding to LDL receptors within the liver and promoting their degradation [107]. Through the reduction in LDL receptors, LDL up take is inhibited and LDL levels increase within the plasma [107]. Higher levels of LDL have been highly associated with cardiovascular risk, and hence PCSK9 has also been associated with higher cardiovascular risk. Librale and team conducted a study in 2018 in patients undergoing CEA to determine the association between CAS, PCSK9 and the development of reduced blood flow within the coronary arteries known as acute coronary syndrome (ACS) [59]. The researchers determined that higher levels of PCSK9 could accurately predict if a patient would develop ACS, with area under the curve (AUC) of 0.719. The authors established a cut-off value of >431.3 ng/mL as a predictor of the development of ACS. Regression analysis also determined a hazard ratio of 17.04 for the risk of ACS in patients with high serum PCSK9 [59].

#### 3.2.4. Paraoxonase-1

Paraoxonase-1 (PON1) is a high-density lipoprotein-associated esterase produced within the liver and secreted into plasma, which is capable of hydrolyzing a wide variety of molecules including lactones, thiolactones, organophosphate pesticides and more [108]. It also has anti-oxidant and anti-inflammatory properties by reducing the oxidation of both HDL and LDL, which are known contributors to the progression of atherosclerosis [109]. Hence, it is hypothesized that PON1 plays a protective role in atherosclerotic disease, with reduced levels demonstrating higher atherosclerotic events [110]. In a group of 74 patients with CAS (of which 43 were asymptomatic), PON1 activity was significantly lower in the symptomatic group than the asymptomatic group (4.6 ± 1.36 versus 5.3 ± 1.19 ng/mL, respectively, *p* = 0.025) with an AUC of 0.654 [18].

### 3.3. Haemostasis

Haemostasis is the process by which multiple interlinked blood components lead to the cessation of bleeding. This process forms a ‘haemostatic plug’ that is composed of several proteins and cells, including platelet aggregates, von Willebrand factor and a fibrin mesh, that bind to and plug the injured area and prevent blood loss [111]. The process can be split into two broad pathways: (1) platelet aggregation, where platelets activate and aggregate to form a plug, and (2) coagulation, which leads to the cleavage of soluble fibrinogen to non-soluble fibrin that binds to activated platelets to strengthen the haemostatic plug [112]. Increased coagulation and platelet activation are a common complication in patients with CAS [113,114]. Narrowing of the carotid artery due to atherosclerotic plaque can lead to turbulent blood flow and shear stress on vascular endothelial lining, leading to further endothelial damage and an increased risk of thrombosis. The following markers of hypercoagulability have been demonstrated in patients CAS.

#### 3.3.1. Plasminogen Activator Inhibitor-1

The plasminogen activator system is a series of activators and inhibitors that regulate fibrinolysis and play important physiological roles in coagulation, inflammation and wound healing [115]. In this cascade, plasminogen is converted to its active form plasmin through the activity of plasminogen activator, which ultimately leads to the degradation of fibrin [116]. The Plasminogen activator inhibitor type 1 (PAI-1), also known as Serpin E1, inhibits the activity on plasminogen activator and prevents the breakdown of fibrin. PAI-1 has been associated with several atherosclerotic diseases, as well as thrombophilia, septicemia and metabolic syndrome. One study demonstrated that PAI-1 is elevated in patients with asymptomatic CAS compared to the symptomatic group (*p* = 0.03) [27]. Similarly, in a small cohort of patients, Sharma and group noted a non-significant trend towards higher levels of PAI in asymptomatic patients [21].

#### 3.3.2. Von Willebrand Factor

Von Willebrand Factor (vWF) is a protein that plays a vital role in hemostasis and is released by vascular endothelial cells and megakaryocytes [117,118]. Under moderate to high shear stress, vWF tethers platelets to the injured vascular endothelium through interactions with collagen. Atherosclerosis can lead to increased shear stress and higher blood flow in areas of atherosclerotic plaque build-up; hence, high vWF levels have been hypothesized to increase the likelihood of thrombotic risk. Several studies demonstrated that higher levels of vWF are significantly associated with adverse outcomes, including death, in patients with pre-existing cardiovascular disease [119]. A study of 811 patients with CAS demonstrated that patients within the highest quartile of vWF levels (determined through levels of vWF antigen, a protein that highly correlated with vWF levels) have a 2.13 increased risk of a cardiovascular events when compared to the lowest quartile.

#### 3.3.3. Factor VII Activating Protease

Factor VII Activating Protease (FSAP) is a serine protease found within plasma that has significant homology to other fibrinolytic enzymes, and is suspected to play a role within hemostasis. Several substrates have been identified such as factor VII, fibrinogen and tissue factor pathway inhibitor, and also other functions such as contributions to inflammation, endothelial permeability and vascular remodeling have been proposed [120,121,122,123]. A study conducted in 2007 demonstrated that FSAP was higher in patients with unstable coronary artery plaques. As such, Parahuleva et al. conducted a small study in a group of 24 patients undergoing CEA and determined that FSAP was higher in the plasma of patients with symptomatic CAS (*p* = 0.029), and unstable plaque tended to have higher levels of FSAP than stable plaques (*p* < 0.01) [29].

### 3.4. Markers of Cardiovascular and Kidney Function

Impaired renal function can cause a myriad of cardiovascular disease issues and may also be associated with the development and progression of CAS. Kidney disease, as well as other cardiovascular diseases such as coronary artery and peripheral artery disease, are often associated with several risk factors that overlap with that of CAS, including hypertension, hypercholesterolemia and diabetes millitus. In addition, kidney disease may lead to an accumulation of toxins and waste products in the bloodstream, which may contribute to inflammation and oxidative stress in the arterial wall and increase endothelial injury, thereby increasing the risk of plaque formation. Studies demonstrated that patients with reduced kidney function are more likely to have arterial calcification [124]. Regular monitoring of kidney function and early treatment of kidney disease may help to prevent the establishment of CAS and reduce the risk of cardiovascular events such as stroke and heart attack. The following markers of kidney and cardiovascular health have been associated with CAS.

#### 3.4.1. Homocysteine

Homocysteine (Hcy) is a sulfur-containing amino acid that structurally resembles cysteine and is produced through the metabolism of methionine [125]. Hcy is thought to play a role in atherosclerosis through its involvement in inflammation, oxidative stress and endothelial homeostasis through endothelial nitric oxide synthase (eNOS) inhibition [126]. Hcy has been well established as a risk factor for cardiovascular disease [127].

In a study in 2021 by Zhang et al., Hcy was correlated with the degree of carotid stenosis, with levels increasing with increasing severity of CAS [49]. Several studies demonstrated Hcy’s association with CAS [17,20,25]. One study also demonstrated its ability to determine patients with asymptomatic CAS from patients without CAS, with an odds ratio of 2.89 and 1.42 in diabetic and non-diabetic patients, respectively [20]. Hcy has also been shown to have diagnostic capability for CAS, with a cut-off value of >15 μg/mol demonstrating a sensitivity of 69.3% and a specificity of 62.1% [25]. A large trial (the Heart Outcomes Prevention Evaluation (HOPE)-2 trial) demonstrated that Hcy levels could be reduced by a combination therapy of vitamin B6, B12 and folic acid. With previous studies demonstrating a relationship between Hcy and CAS, research on this combination of vitamin B6, B12 and folic acid, and the prevention of CAS, could be warranted.

#### 3.4.2. Lipoprotein(a)

Lipoprotein(a) (Lp(a)) is a low-density lipoprotein that contains an additional apolipoprotein (a) attached to the apolipoprotein (b) by a disulphide bridge [128]. In a non-acute inflammatory state, levels of Lp(a) were shown to be genetically determined and stable throughout an individual’s lifestyle, with approximately 20% of individuals having consistent elevated levels [128,129]. It has been well established as a risk factor for several cardiovascular diseases, and the American Heart Association recommended for Lp(a) testing in those with a family history of premature (<55 years of age) atherosclerotic cardiovascular disease [128]. Though elevated Lp(a) is a well-known cardiovascular risk factor, only two studies investigated the relationship between Lp(a) and CAS. The earliest study was conducted by Kim et al. in 2011, in which they demonstrated that Lp(a) was independently associated with asymptomatic CAS > 50%, and could diagnose CAS with a sensitivity and specificity of 69.3, and 62.1, respectively [25]. A more recent study investigated if there was an increase in major adverse cardiovascular events in patients with CAS was associated with plasma Lp(a). They determined that patients with Lp(a) had a significantly higher odds of a major adverse cardiovascular event when compared with patients with low Lp(a) (OR: 1.69) [54].

#### 3.4.3. Pregnancy-Associated Plasma Protein A (PAPP-A)

Pregnancy-Associated Plasma Protein A (PAPP-A) is a vital protein for the health development of the fetus and functions by regulating insulin-like growth factor availability [130]. Lower levels of PAPP-A have been associated with an increased likelihood of Down syndrome as well as several other fetal abnormalities and pregnancy-related complications [130]. PAPP-A has also been implicated in cardiac disease, with elevated levels in patients with CAD, likely due to the release of PAPP-A as a result of inflammation and tissue damage [131]. Higher PAPP-A has also been shown to increase the likelihood of atherosclerotic plaque instability [132]. A study from 2010 demonstrated that patients with ruptures plaques had significantly higher serum level levels of PAPP-A compared to those with stable plaque (0.112 ± 0.06 mg/mL versus 0.074 ± 0.04 mg/mL, respectively, *p* = 0.047) [28]. They also noted that PAPP-A correlated with plaque inflammation [28].

#### 3.4.4. N-Terminal Pro B-Type Natriuretic Peptide

B-type natriuretic peptide (BNP) is a peptide pre-hormone produced within the ventricular myocardium and, once cleaved into its active form, is important in the balance of sodium ions, peripheral vasodilatation and inhibition of the sympathetic nervous system [133]. BNP is cleaved into the active BNP form, known as N-terminal pro B-type natriuretic peptide (NT pro-BNP), which is biologically inactive. Research demonstrated its relevance as a marker of cardiac disease [134], but it also may be relevant in CAS. NT pro-BNP has been shown to be independently associated with symptomatic CAS, but not with asymptomatic CAS [58]. NT pro-BNP was also shown to have prognostic potential in patients undergoing CEA, as Duschek and team demonstrated a significant increase in long-term mortality in patients with increased levels of pre-operative NT pro-BNP in males (hazard ratio of 2.53 × 10^−4^ per each mg/dL (95% confidence interval [CI] 1.48 × 10^−4^, 3.58 × 10^−4^, *p* < 0.0001) [56].

#### 3.4.5. Neutrophil Gelatinase Associated Lipocalin

Neutrophil-Gelatinase-Associated Lipocalin (NGAL) is a protein most commonly found within the tubular endothelium of the kidney, but it can also be released from immune cells and hepatocytes [135]. NGAL is typically used as a marker of acute kidney injury [136]. NGAL’s association with CAS has been reported in two studies [26,61]. In 2017, it was demonstrated that patients with symptomatic CAS had significantly increased levels of NGAL when compared to asymptomatic patients. Furthermore, all patients with vulnerable plaques were noted to have increased NGAL levels [61]. In a study published in 2019 with a small group of asymptomatic patients, NGAL was found to not correlate with CAS severity but NGAL was significantly increased in asymptomatic patients with vulnerable plaques [26].

### 3.5. Bone Health

There is evidence to suggest that certain proteins involved in bone health are associated with the development and progression of CAS. Calcification of atherosclerotic plaque has been suggested to be histomorphologically similar to bone [137,138]. In atherosclerotic disease, calcification occurs through the deposition of minerals, such as calcium, into the vascular tissue, which can harden atherosclerotic plaque, and stiffen the vessel, further impeding optimal blood flow [124]. More research is needed to fully understand the relationship between bone health proteins and CAS, as well as novel treatments and prevention of atherosclerotic calcification. The following are three proteins involved in bone health and remodeling that have been demonstrated to be associated with CAS.

#### 3.5.1. Osteoprotegrin

Osteoprotegrin (OPG) is a member of the TNF receptor family and acts as a decoy receptor for the receptor activator of nuclear factor kappa-Β ligand (RANKL), a protein that activates osteoclasts and leads to bone resorption [139]. Hence, OPG prevents bone resorption; however, it can also act as a cytokine and can have implications on the calcification of the vasculature [140]. Previous studies in OPG knockout mice demonstrated significant arterial calcification. As such, newer studies posited its possibility as a marker for atherosclerotic disease [140]. Kwon et al. previously categorized CAS patients with calcified plaque (CP), non-calcified plaque (NCP) and no plaque controls (NP). The researchers determined that patients in the CP group had significantly elevated levels of OPG, whereas those in the NCP and NP group had no significant difference in OPG levels (median [interquartile range] = 4016 [1410], 3210 [1802] and 3204 [1754] pg/mL, respectively, *p* < 0.05). Elevated levels of OPG were significantly correlated with calcification of plaque, with an odds ratio of 1.5 [35].

#### 3.5.2. Osteopontin

Osteopontin (OPN) is a phosphorylated sialoprotein, primarily found within the mineralized extracellular matrix of bone and teeth, and inhibits the formation of hydroxyapatite [141,142]. This protein, however, can also be found in a wide range of organs and tissues, such as the kidney, smooth muscle cells, as well as within plasma, suggesting a wide range of functions [143]. In smooth muscle cells, it is known to be released in response to stress, as well as in response to inflammation [143]. In a previous study, researchers determined that in patients with CAS, OPN was highly correlated with other inflammatory proteins, such as neutrophils, total macrophages, lipids and MMP-9 content. They also demonstrated that patients with higher levels of OPN have significantly more major adverse cardiovascular events when compared to CAS patients with lower OPN levels. Based on these data, the authors established a cut-off value of >70 ng/mL of OPN as an independent predictor of MACE independent of age, gender and symptomatic status (AUC 0.750 [95% CI 0.681–0.810], *p* = 0.001) [45].

#### 3.5.3. Orosomucoid

Orosomucoid, or α-1-acid glycoprotein, is produced by hepatocytes and is an acute-phase protein whose overall function is relatively unknown; however, it is understood to be a carrier protein for charged lipophilic compounds, anionic drugs and steroids in plasma [144]. It is associated with angiogenesis, and its expression can be induced by other cytokines, such as TNF. Previous studies demonstrated the orosomucoid can alter platelet shape and may contribute to platelet activation [145]. One retrospective analysis conducted by Berntsston et al. in 2016 investigated the relationship between orosomucoid, carotid plaque and stroke incidence and determined that Orosomucoid was significantly higher in subjects with carotid plaque when compared to those without carotid plaque. They also noted that when adjusted for age and sex, Orosomucoid was significantly associated with strokes (HR: 2.13) [44]

### 3.6. Cellular Structure

Atherosclerotic plaque build-up that occurs in CAS can cause significant changes to the cellular structure of the endothelial lining, smooth muscle cells, as well as significant changes to the vasculature within the carotid artery [146]. Plaque build-up and calcification also leads to changes within the extracellular matrix, which contribute to the rigidity and reduced compliance of arteries in addition to that caused by plaque and calcification [147]. Two proteins associated with cellular structure have been demonstrated as potential markers of CAS.

#### 3.6.1. Matrix Metalloproteinases

Matrix Metalloproteinases (MMPs) are a family of enzymes that can lead to the degradation of almost all proteins found within the extracellular matrix. Their function, however, is still far more complex, playing an important role in angiogenesis, cellular differentiation, apoptosis, wound healing and many more [148]. Though they have been also shown to be associated with other diseases, (e.g., cancer [149]), several studies demonstrated their potential as a marker for CAS. Moreno-Ajona D. et al. showed that MMP-1, 7 and 10 were upregulated in patients with CAS, while also demonstrating that, specifically, MMP-7 could predict adverse events such as myocardial infarctions, stroke and cardiovascular death in patients as well, with a hazard ratio of 1.15 [41]. Several other studies demonstrated similar associations between MMPs and CAS compared to control patients [42,72], and vulnerable plaques [26,61].

#### 3.6.2. Vimentin

Vimentin (VIM) is a cytoskeletal intermediate filament that maintains cellular integrity and shape, and prevents mechanical stress [150]. It is primarily found within mesenchymal cells, but can also be found in other cells with distinct nuclei, as it anchors organelles the nucleus, endoplasmic reticulum and mitochondria [151]. Vimentin over-expression has been linked to several cancers, including prostate cancer, central nervous system tumors and breast cancer [152]. Vimentin was previously shown to be associated with carotid artery disease, with increasing serum Vimentin correlating with the number of diseased vessels [153]. Previously, research studied the levels of Vimentin in patients with CAS with a follow-up period of 22 years. The authors determined that patients within the 1st quartile of serum vimentin levels were at a 1.47-times increased risk of suffering from an ischemic stroke when compared to the fourth quartile [53].

### 3.7. Growth Factors

Growth factors are proteins that play important roles in cell growth, proliferation and differentiation throughout development and into adulthood. There are many different growth factors that play various roles in physiological processes in the development and maintenance of the cardiovascular system, and in the initiation and progression of atherosclerotic disease. Some growth factors may have detrimental effects that lead to further progression of the atherosclerotic plaque; however, some may be beneficial, such as those that promote angiogenesis and vascular remodeling. A common manifestation of early atherosclerosis is the migration and proliferation of immune cells, and smooth muscle cells, as well as platelet activation that are, in part, induced through the release of local growth factors. Below are three growth factors that are implicated in the etiology of CAS.

#### 3.7.1. Fibroblast Growth Factor—23

Fibroblast growth factor 23 (FGF23) is a protein that belongs to the fibroblast growth factor family, a family of proteins that have a wide range of function, primarily relevant in development processes, such as development of the brain, angiogenesis and limb development [154]. FGF23, specifically, is produced by osteoblasts and osteocytes within the bone marrow, and inhibits the resorption of phosphate from bone, and reduces the production of 1α,25-dihydroxyvitamin D3 (Vitamin D) in the kidneys further reducing phosphate levels [155]. Previous studies demonstrated that abnormalities in the levels of FGF23 lead to the phosphate wastage and high levels of phosphate excretion in urine [156]. A study conducted by Del Porto F et al. investigated FG23 levels in patients with CAS. The 35 patients recruited were split into two groups; patients with complicated and uncomplicated plaques. The authors noted that patients with complicated plaque had significantly increased serum levels and plaque expression of FGF23 (*p* < 0.05). No significant difference was observed in serum levels of FGF23 when comparing patients with uncomplicated plaques versus healthy controls [46].

#### 3.7.2. Hepatocyte Growth Factor

Hepatocyte growth factor (HGF) is a hepatic protein known for its importance in development and angiogenesis, and has been shown to not only function as a hepatocyte mitogen but influence cell motility, morphology and apoptosis [157]. Due to its influence on angiogenesis, a study from 2010 investigated HGF levels in patients with CAS. The researchers noted a significant increase in neovascularization in patients with symptomatic CAS than asymptomatic patient and also observed significant higher HGF levels in both plaque and serum of patients with symptomatic disease (*p* = 0.002) [32].

#### 3.7.3. Platelet Derived Growth Factor

Platelets were demonstrated to play a highly complex role in both the initiation and progression of atherosclerotic disease, as well as complications, such as thrombus formation [158]. Platelet-derived growth factor (PDGF) is a protein released from activated platelets that activates monocytes, macrophages and fibroblasts, leading to chemotaxis and proliferation [159]. PDGF also stimulates the proliferation of vascular smooth muscle cells into atherosclerotic areas of the vasculature, which may lead to plaque stability [160]. Previously, it has been demonstrated that patients with symptomatic CAS had reduced serum levels of PDGF when compared to patients with asymptomatic CAS (*p* = 0.001) [32].

### 3.8. Hormones

Hormones are protein messengers produced by endocrine glands that are released into circulation and have effects on physiological processes across the body. Hormones can lead to large scale physiological changes in processes such as energy metabolism, hunger, inflammation, angiogenesis and more. Hormones effecting these processes can lead to changes in atherosclerotic plaque formation, such as reducing inflammation and leading to reduced plaque progression, or increasing cellular proliferation. The following five hormones have been postulated to play a role in CAS, and have the potential to be biomarkers for the disease.

#### 3.8.1. Adiponectin

Recently, the influence of perivascular adipose tissue on the modulation of vascular smooth muscle cells, cell signaling and vascular remodeling has been well established [161]. Adiponectin is the most highly secreted protein by adipose tissue [162]. It has known functions in energy metabolism, through the promotion of fatty acid oxidation, insulin sensitization and improved glucose metabolism [163]. It was also shown to reduce atherosclerosis and inflammation [162]. Within the literature, there seems to be a lack of agreement between findings for adiponectin’s use as a biomarker for CAS. Serum levels of adiponectin have been demonstrated to be reduced in patients with vulnerable carotid plaques when compared to stable plaque patients, suggesting a reduction in anti-inflammatory cytokines [47]. In another study, no differences were observed when comparing stable and unstable plaques [51]. Sharma et al. demonstrated that patients with symptomatic CAS had higher levels of adiponectin in perivascular tissue when compared to asymptomatic patients, but this was not reflected within plasma levels [21]. Lastly, in one study from 2012, the authors determined that higher levels of adiponectin were independently associated with all-cause mortality in patients undergoing CEA (hazard ratio 1.46 m 95% confidence interval [CI], 1.14–1.86) [64].

#### 3.8.2. Leptin

Leptin is a hormone released from adipose tissue and has well-known functions in appetite regulation through the modulation of satiety. A reduction in leptin levels (or leptin sensitivity) initiates a cascade of events that lead to the “starvation” response (decrease sympathetic nervous system activation, reduced energy expenditure, increased hunger, etc.) [164]. Two studies analyzed the relationship between leptin levels and CAS. One study demonstrated that patients with symptomatic CAS had reduced levels of leptin when compared to patients with no symptoms (7.1 ± 1.3 versus 14.4 ± 4.7 ng/dL, respectively, *p* < 0.001) [43]. Another found no difference in circulating leptin levels in patients with CAS nor a significant correlation between leptin levels and overall plaque stability, although levels of leptin were increased in patients with some specific histological features of plaque instability, such as plaques with inflammatory cell cap infiltration, and lipid cores [51].

#### 3.8.3. Resistin

Resistin is an inflammatory regulator that stimulates the release of several inflammatory cytokines such as TNF-α, IL-t and MCP-1 from human peripheral blood mononuclear cells, macrophages and vascular cells [165]. Resistin is involved in obesity-induced insulin resistance within mice, but its role in human insulin resistance is contested, though it does seem to play a role in type-2 diabetes mellitus, obesity and its related cardiovascular risk factors [166,167,168]. Two recent studies investigated the levels of resistin in relation to carotid artery stenosis. Jurin et al. demonstrated significantly higher levels, both within plasma and carotid plaque expression, of resistin in patients with unstable carotid plaque. Resistin levels were also increased in symptomatic patients with ischemic stroke when compared to asymptomatic patients (*p* < 0.001), and elevated levels of resistin also predicted increased risk of cerebral symptomology (odds ratio 1.237, 95% confidence interval [CI] 1.079–1.420, *p* = 0.002) [55]. In contrast, another study found no significant association between resistin levels and plaque instability, with resistin only significantly elevated in symptomatic patients relative to asymptomatic patients with type-2 diabetes mellitus [51].

#### 3.8.4. Chromogranin

Chromogranin A (CgA) is a pro-hormone released by neuroendocrine cells and functions by regulating vascular physiology and angiogenesis. Chromogranin A (CgA), the full-length CgA (CgA1-439) and its fragment CgA1-76 (called vasostatin-1, VS-1) preserve the physiological integrity of the endothelial barrier and are antiangiogenic, whereas CgA1-373 is proangiogenic [169]. Previously, CgA testing has been used to measure the amount of CgA in the blood but has been typically reserved for the diagnosis and management of patients with neuroendocrine tumors [170,171]. One study noted plasma levels of VS-1 and total-CgA significantly correlated with carotid artery maximum stenosis, and regression analysis indicated it was a predictor of maximum stenosis even after adjustment of confounders. They also noted higher levels of CgA1-439 were predictive of hypoechogenic plaque (i.e., plaques with higher levels of fibrous tissue and calcification), which has been associated with higher risk of adverse events [34,172,173].

#### 3.8.5. Nesfatin-1

Nesfatin-1 is a novel neuropeptide expressed in the hypothalamus, in other areas of the brain, in pancreatic islets, gastric endocrine cells and adipocytes [174]. Nesfatin-1 has been identified in regulating hunger and fat storage in mammals where an increase in nesfatin-1 levels in the hypothalamus decreased the hunger sensation resulting in a potential loss of body fat and weight. Nesfatin-1 has also been shown to have a role in regulating blood pressure, heart rate and cardiomyocyte metabolism. Nesfatin-1 was recently found to increase sympathetic activity leading to an increase in mean arterial pressure [175]. In a study conducted by Dai et al., nesfatin-1 levels were found to be lower in patients presenting with acute myocardial infarction when compared to normal individuals. They also demonstrated an association between serum nesfatin-1 concentrations and the development and severity of peripheral artery disease in patients with diabetes mellitus [176].

Previously, it has been reported that serum nesfatin-1 levels were negatively correlated with the rate of CAS. Nesfatin-1 levels were lower in patients with CAS ≥ 60% when compared with CAS < 60%. Nesfatin-1 levels were higher in healthy controls. The study established that low levels of nesfatin-1 levels is an independent risk marker for carotid artery disease and the plaque morphology revealed that nesfatin-1 levels were lower in the non-calcified plaque group than in the calcified plaque group [22].

## 4. Discussion

Atherosclerotic plaque within the carotid artery, known as carotid artery stenosis, can be influenced by processes such as bone health, inflammation and immunity, extracellular matrix remodeling and many more. CAS is a common manifestation of atherosclerotic disease that can cause debilitating consequences in patients. CAS can cause transient ischemic attacks (TIA) and cerebrovascular attacks (CVA), which are known to be the second most common cause of death and a major cause of disability worldwide [177]. Early diagnosis of CAS is vital in order to provide adequate prevention strategies to reduce the progression of the disease and to reduce the risk of adverse cardiovascular events [178]. The current best medical management of CAS includes hypertension, hypercholesterolemia, and diabetes management, cessation of smoking and antiplatelet regimes; however, despite this management, many patients will still be affected by adverse cardiovascular events.

Patients with carotid artery stenosis can be categorized into two broad categories, patients who have some degree of carotid artery atherosclerotic plaque, but are asymptomatic and have had no associated neurological symptoms, and symptomatic patients who have had a recent TIA, CVA or other neurological symptoms related to stenosis of the carotid artery [179]. Medical treatments and interventions are different depending on the degree of carotid stenosis, as well as symptomology. Patients with symptomatic CAS or asymptomatic CAS with low risk for surgery are recommended to undergo carotid endarterectomy, or carotid artery stenting in certain circumstances [14]. Asymptomatic patients can have up to 99% stenosis of their carotid artery with having no symptoms, yet placing them at higher risk of TIA and CVA than those without CAS. There exist few accepted methods to diagnose asymptomatic CAS apart from ultrasound imaging such as carotid Doppler ultrasound. Consequently, biomarkers for CAS are critically needed to aid with earlier diagnosis for CAS in routine testing as asymptomatic patients may never demonstrate symptoms until a stroke occurs [180].

There is currently an extensive list of potential markers for the diagnosis of CAS, and some have been shown to be able to distinguish symptomatic patients from asymptomatic patients. Those markers that are elevated in patients with asymptomatic CAS, such as leptin, IL-6 and PON-1, among others, may be extremely beneficial to flag those with elevated levels of these markers for further carotid ultrasound follow-up. Carotid plaques, once identified, can be classified following AHA guidelines, based on plaque morphology identified during ultrasound, into two groups: stable plaques and vulnerable plaques [181]. Stable plaques are characterized by small lipid cores and large fibrous caps, whereas unstable plaques are characterized by thin fibrous caps, larger altered lipid cores and the potential of intra-plaque thrombi and fibrous cap ruptures. Plaque morphology can play a significant role in the pathophysiology of CAS, with those with unstable and non-calcified plaque having higher risk of plaque rupture and adverse events compared to those with stable and calcified plaque [182]. Several of the demonstrated biomarkers are also able to predict plaque morphology in patients with CAS, such as nesfatin-1, NGAL, MMP-9/NGAL, PAPP-A, HMGB1 and osteoprotegerin and others. Markers for plaque morphology could be beneficial for risk stratification purposes, disease progression monitoring, and accessibility and cost effectiveness. Similarly, some markers have been demonstrated to have prognostic value in predicting adverse cardiovascular events in CAS patients, including hsCRP, adiponectin, VWF:Ag and PCSK9, and many more determined in this review. These markers may be valuable for the early diagnosis of CAS, as well as for risk stratification in order to prescribe more rigorous preventive medical management.

Recently, artificial intelligence and machine learning have become a hot topic in the diagnosis and prognostication of many diseases. Recent studies used several biomarkers in combination to create more robust diagnostic and prognostic tools for physicians with stronger sensitivity and specificity than one marker alone [183]. The use of machine learning models, such as decision trees and random forests, for CAS may provide a method for better diagnostics and risk stratification by integrating risk scores for multiple protein biomarkers, while also adjusting for the contribution of age, sex, past medical history and other confounders that are associated with disease initiation and progression. These models can be trained, based on these biomarkers and cardiovascular risk factors, to determine the likelihood of each patient either having the disease or not, or stratifying high risk patients more likely to suffer from adverse cardiovascular events from lower list patients. This empowers physicians with a tool to assess the risk of CAS, and subsequently tailor the aggressiveness of medical treatment required. There are currently no strong markers for CAS that have been clinically validated that can be used for either diagnose or prognostics alone and, hence, this comprehensive review demonstrated several markers that could be used in an integrative approach with machine learning to create a panel of markers for CAS. Those interested in this approach can choose proteins from this review based on their strengths in what specifically the researchers are hoping to achieve, such as stroke prediction, or diagnosing asymptomatic patients or predicting plaque morphology. With this review, several potential markers were outlined that can be integrated into machine learning models and diagnostic and prognostic markers for CAS.

## 5. Conclusions

In this comprehensive literature review, we outlined several biomarkers, covering a wide range of physiological responses and processes, that are relevant to the diagnosis and/or prognosis of CAS. There is yet to be a clinical biomarker for CAS that is robust, with widespread acceptance as clinically relevant. Integrating a few of these biomarkers, in combination with cardiovascular risk factors, into a computational machine learning model may be a beneficial and accurate method for diagnosing patients with CAS, predicting outcomes in patients, or determining plaque morphology. This will also allow for better risk stratification of patients with CAS, and allow for earlier medical management to prevent the devastating adverse events related to CAS.

## Figures and Tables

**Table 1:** Current available research on plasma and serum protein biomarkers for carotid artery stenosis, obtained through MEDLINE search between 2010 and 2022.

Title	Authors	Patients and Sample Size	Findings
Metabolomics Study in Severe Extracranial Carotid Artery Stenosis [17]	Tsong-Hai L. et al., 2019	130 male patients with at least one carotid artery stenosis ≥ 60%	CAS group had significantly lower levels of total cholesterol and high-density lipoprotein than healthy controls (*p* < 0.001). Homocysteine levels in CAS patients were higher than that in controls (*p* = 0.011)
Paraoxonase-1 and Symptomatic Status in Carotid Artery Disease [18]	Lioudaki S. et al., 2020	74 patients undergoing CEA	Symptomatic CAS patients differed significantly in PON-1 levels compared to asymptomatic patients, with the symptomatic patients having lower PON-1 levels (5.3 ± 1.19 vs. 4.6 ± 1.36 ng/mL; *p* = 0.025). The AUC from ROC analysis was 0.654 (*p* = 0.023)
Increased Serum CRP-Albumin Ratio Is Independently Associated with Severity of Carotid Artery Stenosis [19]	Yildirim T. et al., 2020	269 patients undergoing carotid angiography, split into group 1 (stenosis < 70%, n = 189) or group II (stenosis ≥ 70%, n = 80)	Group 2 had higher C-reactive protein to albumin ratio (CAR) compared to group 1 (0.56 ± 0.25 vs. 0.14 ± 0.01, *p* < 0.001). ROC curve demonstrated an AUC for the CAR to predict severe CAS was 0.798 (95% CI: 0.741–0.854, *p* < 0.001). CAR was an independent risk factor of severe CAS.
Homocysteine and its Relationship to Asymptomatic Carotid Stenosis in a Chinese Community Population [20]	Jia J. et al., 2016	5393 participants, 361 of who were diagnosed with asymptomatic CAS	Asymptomatic CAS patients had higher Hcy than healthy controls. A cut of Hcy > 19.3 mumol/L independently predicted CAS (OR 2.89 (1.02–8.22) in DM vs. 1.42 (0.95–2.12) in non DM)
Perivascular Adipose Adiponectin Correlates with Symptom Status of Patients Undergoing Carotid Endarterectomy [21]	Sharma G. et al., 2015	34 patients with CAS (19 asymptomatic and 15 symptomatic)	Symptomatic CAS patients had 1.9-fold higher adiponectin levels compared to asymptomatic patients (*p* = 0.005).
The Relationship Between Nesfatin-1 and Carotid Artery Stenosis [22]	Kuyumcu A. et al., 2018	Patients with no atherosclerotic plaques (n = 60), CAS < 60% (n = 60), and CAS ≥ 60% (n = 60).	Nesfatin-1 was lower in the CAS <60% group compared to healthy controls (*p* < 0.001). Serum nesfatin-1 levels were further reduced in the CAS ≥ 60% group versus the CAS < 60% group (*p* < 0.001). Serum nesfatin-1 levels were independently associated with CAS. Calcified plaque had higher nesfatin-1 levels compared no non-calcified plaque.
Increased Levels of Legumain in Plasma and Plaques from Patients with Carotid Atherosclerosis [23]	Lunde N.N. et al., 2017	254 patients with CAS	Median plasma legumain levels were higher in patients with CAS compared to healthy controls (median 2.0 versus 1.5 ng/mL, respectively; *p* = 0.003),
Levels of circulating neopterin in patients with severe carotid artery stenosis undergoing carotid stenting [24]	Chen Y.L. et al., 2014	50 patients with severe CAS undergoing stenting, age- and gender-matched acute ischemic stroke patients (n = 120) and control subjects (n = 33)	Neopterin was significantly higher in patients with CAS when compared to both ischemic stroke and healthy control patients (*p* < 0.001). Neopterin was significantly higher in ischemic stroke patients than controls (*p* < 0.001).
Biomarkers of Asymptomatic Carotid Stenosis in Patients Undergoing Coronary Artery Bypass Grafting [25]	Kim S.J. et al., 2011	757 patients undergoing preoperative carotid artery duplex scanning	Lipoprotein(a), and homocysteine were independently associated with carotid stenosis of ≥ 50%. Odds ratio for lipoprotein(a), and homocysteine comparing the highest and lowest quartile was 2.17 (1.16 to 4.05), and 2.13 (1.20 to 3.79), respectively.
Neutrophil Gelatinase Associated Lipocalin (NGAL) for Identification of Unstable Plaques in Patients with Asymptomatic Carotid Stenosis [26]	Eilenberg W. et al., 2019	83 patients with asymptomatic CAS	CAS patient with vulnerable plaques showed highest levels of NGAL and MMP-9/NGAL complex (*p* = 0.0003 and *p* = 0.0078, respectively).
Significance of vitronectin and PAI-1 activity levels in carotid artery disease: comparison of symptomatic and asymptomatic patients [27]	Ekmekci H. et al., 2013	37 patients with CAS	PAI-1 activity levels were higher in asymptomatic CAS patients compared to symptomatic patients (*p* = 0.038). PAI-1 and vitronectin were also positively correlated in symptomatic CAS patients (r = 0.399, *p* = 0.039).
Is serum pregnancy-associated plasma protein A really a potential marker of atherosclerotic carotid plaque stability? [28]	Heider P. et al., 2010	66 CAS patients (29 asymptomatic and 37 symptomatic)	PAPP-A was significantly higher in CAS patients with unstable plaques when compared to stable plaques (0.10 ± 0.06 vs. 0.07 ± 0.04 microg mL^−1^, *p* = 0.047). Asymptomatic patients also had higher levels of PAPP-A compared to symptomatic patients (0.11 ± 0.05 vs. 0.069 ± 0.09 microg mL^−1^, *p* = 0.025).
Factor VII Activating Protease Expression in Human Platelets and Accumulation in Symptomatic Carotid Plaque [29]	Parahuleva M.S. et al., 2020	24 CAS patients (14 asymptomatic and 10 symptomatic)	FSAP levels were higher in symptomatic patients when compared to asymptomatic patients, both in plasma and carotid plaque.
Association between carotid plaque vulnerability and high mobility group box-1 serum levels in a diabetic population [30]	Biscetti F. et al., 2021	873 diabetic patients, including 347 patients with CAS who underwent carotid endarterectomy and 526 diabetic patients without CAS	HMGB1 serum levels, osteoprotegerin, high-sensitivity C-reactive protein, tumor necrosis factor-alpha and interleukin-6, were significantly elevated in CAS patients with diabetes when compared to diabetic patients without CAS. HMGB1 and osteoprotegerin were independently associated with unstable plaque.
Circulating serum fatty acid synthase is elevated in patients with diabetes and carotid artery stenosis and is LDL-associated [31]	De Silva G. et al., 2019	13 CAS patients with DM, 13 patients with CAS and no DM, 13 controls	cFAS levels were higher in CAS when compared to controls (*p* < 0.01). Patient with diabetes had higher cFAS than non-diabetic patients.
A comparative study of carotid atherosclerotic plaque microvessel density and angiogenic growth factor expression in symptomatic versus asymptomatic patients [32]	Chowdhury M. et al., 2010	16 CAS patients (8 asymptomatic and 8 symptomatic) and 6 healthy age-matched controls.	HGF levels were higher in symptomatic CAS patients compared to asymptomatic CAS patients and healthy controls (*p* = 0.002). PDGF levels were lower in symptomatic CAS patients compared to asymptomatic CAS patients (*p* = 0.036).
Increased circulating chemerin in patients with advanced carotid stenosis [33]	Kammerer A. et al., 2018	178 patients prior to carotid endarterectomy (CEA) and 163 age- and gender-matched controls	Chemerin levels were elevated in CAS patients compared to healthy controls (*p* < 0.001). There was no significant difference in Chemerin levels in asymptomatic CAS patients compared to symptomatic CAS patients.
Plasma levels of vasostatin-1, a chromogranin A fragment, are associated with carotid artery maximum stenosis: A pilot study [34]	Bachetti T. et al., 2017	81 patients with asymptomatic CAS	CgA levels correlated with CAS severity (r = 0.349, *p* = 0.001 and r = 0.256, *p* = 0.021, respectively). Chromogranin fragment significantly predicted CAS severity (regression coefficient = 12.42, SE = 4.84, *p* = 0.012).
Serum Osteoprotegerin Is Associated With Calcified Carotid Plaque: A Strobe-Compliant Observational Study [35]	Kwon A. et al., 2016	145 patients with CAS	Osteoprotegerin was significantly higher in CAS patients with carotid plaque when compared to healthy controls (*p* < 0.05).
Serum lipopolysaccharide-binding protein as a marker of atherosclerosis [36]	Serrano M. et al., 2013	332 patients with CAS	LBP was associated with CAS intimal medial thickness (r = 0.27, *p* < 0.0001). LBP was f significantly higher in patients with carotid plaque (n = 50; 32.7 ± 12.5 vs. 28.7 ± 10.7; *p* = 0.021).
High Levels of S100A12 Are Associated With Recent Plaque Symptomatology in Patients With Carotid Atherosclerosis [37]	Abbas A. et al., 2012	159 patients with CAS 22 healthy control	Calgranulins S100A12 was higher in patients with CAS compared to healthy control subjects.
Circulating lipoprotein-associated phospholipase A2 in high-grade carotid stenosis: a new biomarker for predicting unstable plaque [38]	Sarlon-Bartoli G. et al., 2012	42 patients with CAS	CAS patients with unstable plaque had significantly higher median levels of Lp-PLA2 compared to CAS patients with stable plaque (222.4 (174.9–437.5). Lp-PLA2 was higher in asymptomatic CAS patients with unstable plaque (226.8 ng/mL (174.9–437.5) when compared to asymptomatic CAS patients with stable plaque (206.9 ng/mL (174.9–270.6) (*p* = 0.16).
Variance in Biomarker Usefulness as Indicators for Carotid and Coronary Atherosclerosis [39]	Pearl M. et al., 2016	522 patients, 77 of which had CAS > 50%	Patients with significant carotid stenosis had higher levels of hs-CRP (9.4 ± 17 vs. 6.3 ± 13 mg/L, *p* = 0.001 compared to healthy controls
The value of C-reactive protein in symptomatic versus asymptomatic carotid artery stenosis [40]	Guven H. et al., 2013	48 patients with CAS (16 asymptomatic and 21 symptomatic patients) and 22 healthy controls	Hs-CRP was significantly elevated in CAS patient, both symptomatic (*p* = 0.001) and asymptomatic (*p* < 0.001) patients compared to healthy controls. There was no significant difference between CAS patients with asymptomatic and symptomatic disease.
Elevated circulating metalloproteinase 7 predicts recurrent cardiovascular events in patients with carotid stenosis: a prospective cohort study [41]	Moreno-Ajona D. et al., 2020	31 patients with CAS	MMP-1, 7 and 10 were significantly elevated in patients with CAS compared to healthy controls. Circulating MMP-7 was an independent predictor of CAS (HR = 1.15 *p* = 0.006).
MMP-12 and TIMP Behavior in Symptomatic and Asymptomatic Critical Carotid Artery Stenosis [42]	Del Porto F. et al., 2017	40 patients with CAS (30 asymptomatic, and 10 symptomatic patients) and 31 controls	MMP-12 was elevated and TIMPS was decreased in patients with CAS. TIMPS levels were higher in patients with symptomatic CAS compared to asymptomatic CAS patients.
Serum leptin levels in patients undergoing carotid endarterectomy: a pilot study [43]	Bountouris I. et al., 2010	74 patients undergoing CEA with >70% stenosis	Leptin levels were decreased in symptomatic CAS patient s compared to asymptomatic CAS patients. Interleukin-6 levels were elevated in symptomatic patients. Leptin and IL-6 were independent predictors of symptomatic CAS.
Orosomucoid, Carotid Plaque, and Incidence of Stroke [44]	Berntsson J. et al., 2016	4285 subjects without cardiovascular disease undergoing carotid ultrasound	Patient with carotid plaque has significantly higher levels of Orosomucoid compared to healthy controls (mean ± SD: 0.72 ± 0.22 versus 0.69 ± 0.20 g/L; *p* < 0.001). Orosomucoid was associated with stroke with a HR of1.48 comparing the highest and lowest tertile.
Serum levels of osteopontin predict major adverse cardiovascular events in patients with severe carotid artery stenosis [45]	Carbone F. et al., 2018	225 patients with CAS (185 asymptomatic and 40 symptomatic patients)	OPN was significantly increased (almost 2-fold), in patients with symptomatic CAS. OPN levels > 70 ng/mL was significantly associated with MACEs at a 24-month.
FGF-23 levels in patients with critical carotid artery stenosis [46]	Del Porto F. et al., 2015	35 patients with severe CAS	FGF-23 is significantly elevated in CAS patients with complicated plaque compared to patients with non-complicated plaque at first follow-up (*p* < 0.05) and second follow up (*p* = 0.0047).
Inflammatory biomarkers in atherosclerosis: pentraxin 3 can become a novel marker of plaque vulnerability [47]	Shindo A. et al., 2014	58 patients with CAS	PTX3 levels CAS patients with vulnerable plaque when compared to patients with stable plaque.
Lipoprotein Associated Phospholipase A2 as a Marker of Vulnerable Atherosclerotic Plaque In Patients With Internal Carotid Artery Stenosis [48]	Stefanic P. et al., 2017	70 patients with CAS (40 asymptomatic and 30 symptomatic patients)	There were significantly higher levels of Lp-PLA2 (*p* < 0.001) in CAS patients with soft plaque versus patients with stable plaque.
Lp-PLA2 evaluates the severity of carotid artery stenosis and predicts the occurrence of cerebrovascular events in high stroke-risk populations [49]	Zhang F. et al., 2021	823 patients at a high risk of stroke	Lp-PLA2 was significantly higher in CAS patients compared to healthy controls, and was also elevated in patients who had an adverse event compared to patients with no events (*p* < 0.05). Lp-PLA2 was also positively correlated with the degree of CAS (r = 0.093, *p* = 0.07). When comparing the highest and lowest quartiles for LP-PLA2 levels, there was 10.170 times higher risk of events for the highest quartile (OR = 10.170, 95% CI 1.302–79.448, *p* = 0.027).
COMP (Cartilage Oligomeric Matrix Protein) NeoepitopeA Novel Biomarker to Identify Symptomatic Carotid Stenosis [50]	Sandstedt et al., 2021	50 symptomatic patients with CAS, 50 patients with stroke without CAS but small plaques, and 50 controls	COMPneo was higher and in patients with CAS compared to controls. COMPneo was independently predictive of CAS
Circulating Chemerin Is Associated With Carotid Plaque Instability, Whereas Resistin Is Related to Cerebrovascular Symptomatology [51]	Gasbarrino K. et al., 2016	165 patient with CAS undergoing CEA	Chemerin and leptin levels were significantly associated with plaque instability. Resistin was significantly higher in symptomatic CAS patients when compared with asymptomatic CAS patients (*p* = 0.001). Higher resistin levels were also associated with increased risk of cerebrovascular symptomatology (adjusted OR 1.264, 95% CI: 1.004–1.594).
Plasma sICAM-1 as a Biomarker of Carotid Plaque Inflammation in Patients with a Recent Ischemic Stroke [52]	Puig N. et al., 2022	64 patients with CAS	Soluble intercellular adhesion molecule-1 (sICAM-1), soluble vascular adhesion molecule-1 (sVCAM-1), and fractalkine (FKN) were independently associated with plaque inflammation (beta = 0.121, 95% CI 0.061–0.181, *p* < 0.001; beta = 0.144, 95% CI 0.012–0.276, *p* = 0.033; beta = 0.136, 95% CI 0.037–0.235, *p* = 0.008).
Circulating Vimentin Is Associated With Future Incidence of Stroke in a Population-Based Cohort Study [53]	Xiao J. et al., 2021	4688 patients with and without CAS	CAS patients in the highest quartile of vimentin had significantly increased risk of stroke compared to the group with lowest quartile (HR, 1.32 [95% CI, 1.02–1.70]). Higher levels of vimentin were also significantly associated with increased occurrence of plaque.
Elevated Lp(a) (Lipoprotein[a]) Levels Increase Risk of 30-Day Major Adverse Cardiovascular Events in Patients Following Carotid Endarterectomy [54]	Waissi, F. et al., 2020	944 patients with CAS undergoing CEA	>137 nmol/L of Lp(a) was significantly associated with an increased risk of 30-day MACE after CEA.
Association between Circulatory and Plaque Resistin Levels with Carotid Plaque Instability and Ischemic Stroke Events [55]	Jurin I. et al., 2018	78 patients with CAS (38 asymptomatic and 40 symptomatic patients)	Resistin was significantly higher in CAS patient with unstable plaque (*p* < 0.001) in both serum and within atherosclerotic plaque. Patients with ischemic stroke also had significantly higher resistin levels (*p* < 0.001)
N-terminal pro B-type natriuretic peptide (NT pro-BNP) is a predictor of long-term survival in male patients of 75 years and older with high-grade asymptomatic internal carotid artery stenosis [56]	Duschek N. et al., 2011	205 with asymptomatic CAS undergoing CEA	High NT pro-BNP concentration were significantly associated with increased long-term mortality in male patients.
Inflammatory mediators and cerebral embolism in carotid stenting: new markers of risk [57]	Pini R. et al., 2013	20 patients with CAS	Hs-CRP ≥ 5 mg/L was significantly associated with a higher number of new cerebral lesions [16.2 ± 10.7 vs. 4.3 ± 3.4 for hs-CRP < 5 mg/L (*p* = 0.02).
Circulating Biomarkers Predict Symptomatic but Not Asymptomatic Carotid Artery Stenosis [58]	Fatemi S. et al., 2022	5550 patients without CAS	NT pro-BNP (HR: 1.59; 95% CI: 1.20–2.11), and CRP (HR 1.53; CI: 1.13–1.73) were significantly associated with symptomatic CAS.
Serum PCSK9 levels predict the occurrence of acute coronary syndromes in patients with severe carotid artery stenosis [59]	Liberale L. et al., 2018	189 patients with severe CAS undergoing CEA	PCSK9 could accurately predict patients with asymptomatic CAS (AUC: 0.719 [95% CI 0.649–0.781]). Patients with PCSK9 > 431.3 ng/mL were at a greater risk of ACS occurrence (*p* = 0.0003). This cut off could predict risk of ACS (HR 17.04 [95% CI 3.34–86.81]; *p* = 0.001).
Relationship between ADAMTS4 and carotid atherosclerotic plaque vulnerability in humans [60]	Dong H. et al., 2018	48 patients with CAS undergoing carotid endarterectomy	CAS patients with vulnerable plaque has significantly higher levels of ADAMTS4 in both serum and plaque compared to CAS patients with stable plaque (*p* = 0.004 and *p* = 0.021).
NGAL and MMP-9/NGAL as biomarkers of plaque vulnerability and targets of statins in patients with carotid atherosclerosis [61]	Eilenberg W. et al., 2017	136 patients with CAS	NGAL and MMP-9/NGAL complex levels were significantly higher in CAS patients with vulnerable plaques (*p* < 0.001), and significantly higher in symptomatic CAS patients compared to asymptomatic patients (*p* < 0.001). NGAL was independently associated with symptomatic CAS.
Pro B-type Natriuretic Peptide and Midregional Proadrenomedullin are Associated with Incident Carotid Stenosis During Long Term Follow-up [62]	Fatemi S. et al., 2021	5550 patients without CAS	NT Pro BNP was independently associated with CAS (HR 1.36; 95% CI 1.12–1.65; *p* = 0.002).
Von Willebrand Factor Antigen Levels Predict major adverse cardiovascular events in patients with carotid stenosis of the ICARAS study [63]	Kovacevic K et al., 2019	811 Caucasian patients with CAS	Levels of VWF:Ag predicted future cardiovascular events in patients with CAS. Patients with highest VWF:Ag concentrations has significantly higher rates of cardiovascular events (HR 2.15 (95% CI: 1.46–3.16; *p* < 0.001).
High Plasma Adiponectin Concentration is Associated with All-Cause Mortality in Patients with Carotid Atherosclerosis [64]	Persson J. et al., 2012	292 patients with CAS undergoing CEA	High adiponectin levels were significantly associated with increased mortality (HR per standard deviation (SD) increase in adiponectin: 1.46, 95% CI: 1.14–1.86).
Combined Effects of Inflammatory Status and Carotid Atherosclerosis [65]	Mayer F.J. et al., 2016	1065 patients with asymptomatic CAS	Elevated hsCRP levels were significantly associated with an increased risk of all-cause (adjusted HR per increase of 1 mg/dL of hsCRP levels; 1.47, *p* < 0.001).
Inflammatory Markers in Patients with Internal Carotid Artery Stenosis [66]	Puz P. et al., 2013	65 patients with CAS and 30 healthy controls	Interleukin-6, fibrinogen, ESR and CRP were significantly higher in patients with CAS compared to healthy controls (*p* = 0.001, *p* = 0.009, *p* = 0.036, *p* = 0.009, respectively). CAS patients with unstable plaque had significantly elevated levels of TNF-α, interleukin-6, fibrinogen, and higher CRP values compared to CAS patients with stable plaques (*p* < 0.05).

CAS = Carotid Artery Stenosis, hs-CRP = High Sensitivity—C Reactive Protein; IL-6 = Interleukin-6; TNF = Tissue Necrosis Factor; sVCAM-1 = Soluble Vascular Cell Adhesion Molecule-1; HMGB-1 = High Mobility Group Box-1; LPS = Serum Lipopolysaccharide-Binding Protein; IL-8 = Interleukin-8; PAPP-A = Pregnancy-Associated Plasma Protein A; NGAL = Neutrophil Gelatinase Associated Lipocalin; NT-PRO BNP = N-Terminal Pro B-Type Natriuretic Peptide; FGF-23 = Fibroblast Growth Factor—23; HGF = Hepatocyte Growth Factor; PDGF; Platelet Derived Growth Factor; MMP = Matrix Metalloproteinases; PAI-1 = Plasminogen Activator Inhibitor-1; vWF = Von Willebrand Factor; FSAP = Factor VII Activating Protease; LP-PLA2 = Lipoprotein-Associated Phospholipase A2; cFAS = Circulating Fatty Acid Synthase; PCSK9 = Proprotein Convertase Subtilisin/Kexin Type 9; PON1 = Paraoxonase-1, ADAMTS = A Disintegrin and Metalloproteinase with Thrombospondin Motifs.
